# Tris(methyl 3-oxobutanoato-κ^2^ *O*,*O*′)aluminium(III)

**DOI:** 10.1107/S1600536809049812

**Published:** 2009-11-25

**Authors:** Gururaj M. Neelgund, S. A. Shivashankar, T. Narasimhamurthy, Seik Weng Ng

**Affiliations:** a Materials Research Centre, Indian Institute of Science, Bangalore 560 012, India; b Department of Chemistry, University of Malaya, 50603 Kuala Lumpur, Malaysia

## Abstract

In the title compound, [Al(C_5_H_7_O_3_)_3_], three acac-type ligands (methyl 3-oxobutanoate anions) chelate to the aluminium(III) cation in a slightly distorted AlO_6_ octa­hedral coordination geometry. Electron delocalization occurs within the chelating rings.

## Related literature

For the crystal structure of tris­(acetyl­acetonato)aluminium, see: von Chrzanowski *et al.* (2007 ▶).
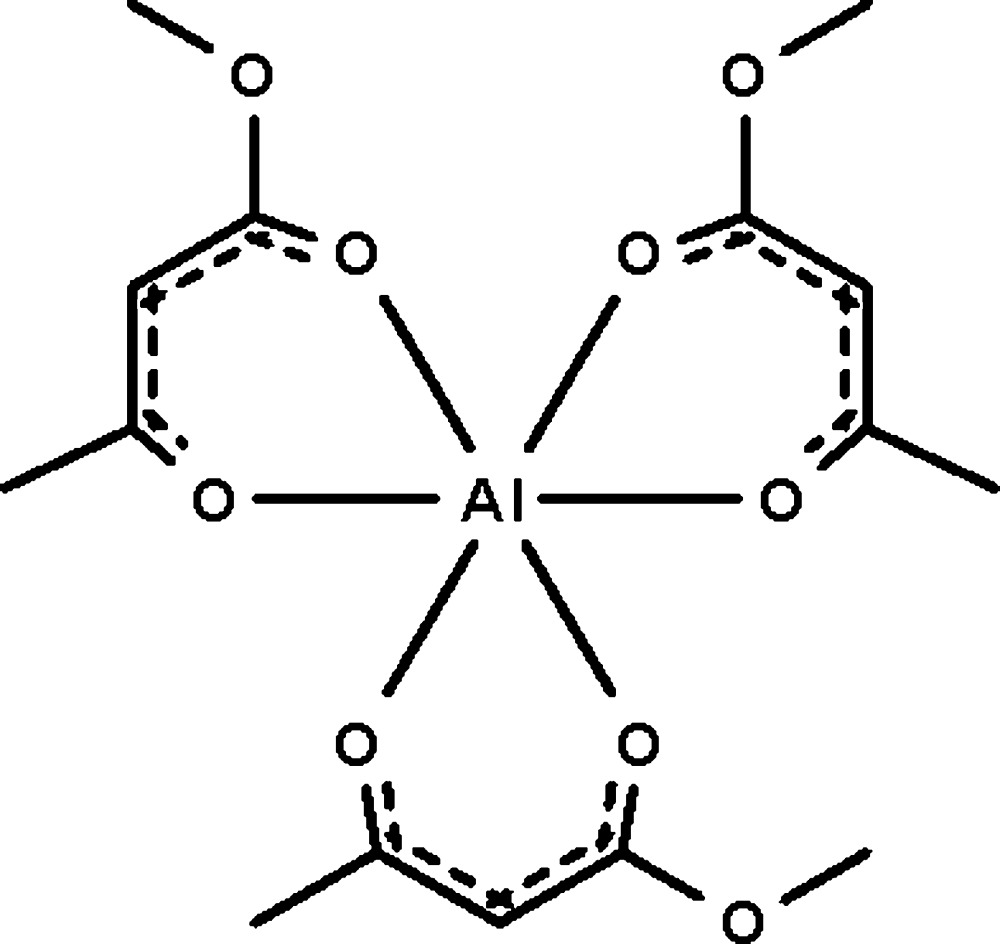


## Experimental

### 

#### Crystal data

[Al(C_5_H_7_O_3_)_3_]
*M* *_r_* = 372.30
Triclinic, 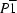

*a* = 6.476 (1) Å
*b* = 9.986 (2) Å
*c* = 14.368 (2) Å
α = 90.478 (2)°
β = 92.229 (2)°
γ = 99.337 (2)°
*V* = 916.1 (2) Å^3^
*Z* = 2
Mo *K*α radiation
μ = 0.15 mm^−1^
*T* = 293 K
0.5 × 0.4 × 0.2 mm

#### Data collection

Bruker SMART APEX diffractometer
Absorption correction: multi-scan *SADABS* (Sheldrick, 1996 ▶) *T*_min_ = 0.927, *T*_max_ = 0.970
8801 measured reflections
3207 independent reflections
2304 reflections with *I* > 2σ(*I*)
*R*_int_ = 0.041

#### Refinement

*R*[*F* ^2^ > 2σ(*F* ^2^)] = 0.068
*wR*(*F* ^2^) = 0.189
*S* = 1.02
3207 reflections
232 parameters
H-atom parameters constrained
Δρ_max_ = 1.26 e Å^−3^
Δρ_min_ = −0.28 e Å^−3^

### 

Data collection: *SMART* (Bruker, 2004 ▶); cell refinement: *SAINT* (Bruker, 2004 ▶); data reduction: *SAINT*; program(s) used to solve structure: *SHELXS97* (Sheldrick, 2008 ▶); program(s) used to refine structure: *SHELXL97* (Sheldrick, 2008 ▶); molecular graphics: *X-SEED* (Barbour, 2001 ▶); software used to prepare material for publication: *publCIF* (Westrip, 2009 ▶).

## Supplementary Material

Crystal structure: contains datablocks global, I. DOI: 10.1107/S1600536809049812/xu2684sup1.cif

Structure factors: contains datablocks I. DOI: 10.1107/S1600536809049812/xu2684Isup2.hkl

Additional supplementary materials:  crystallographic information; 3D view; checkCIF report

## Figures and Tables

**Table 1:** Selected bond lengths (Å)

Al1—O1	1.905 (3)
Al1—O3	1.859 (3)
Al1—O4	1.909 (3)
Al1—O6	1.849 (3)
Al1—O7	1.904 (3)
Al1—O9	1.869 (3)

**Fig. 1.**
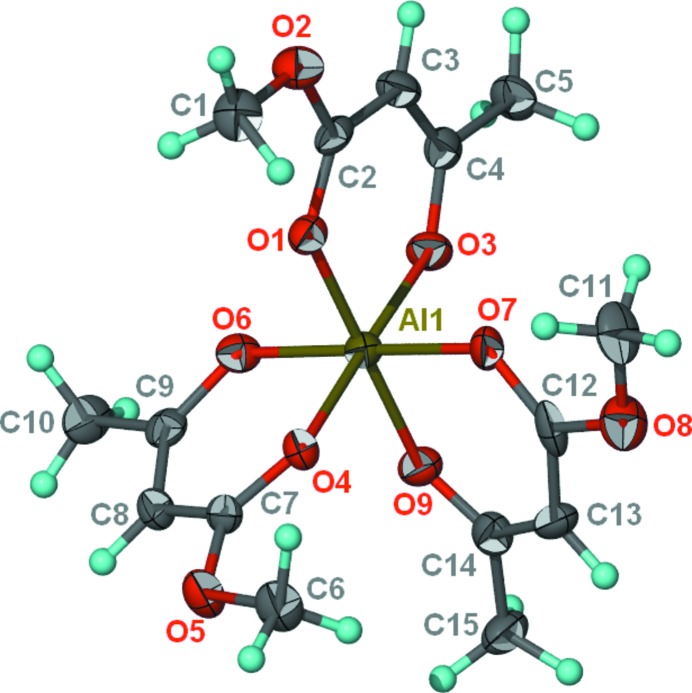
Thermal ellipsoid plot (Barbour, 2001) of Al(C5H7O3)3 at the 50% probability level; hydrogen atoms are drawn as spheres of arbitrary radius.

## supplementary crystallographic information

### Experimental

Aluminium isopropoxide (10 mmol, 2.04 g) was dissolved in toluene (25 ml) under a nitrogen atmosphere. Methyl acetoacetate (30 mmol, 3.2 ml) was added. The mixture turned yellow. The solution was stirred for 6 h. The solvent was removed by fractional distillation under vacuum to yield the product, which was purified by repeated recrystallization from cyclohexane.

### Refinement

Carbon-bound H-atoms were placed in calculated positions (C—H 0.93 to 0.96 Å) and were included in the refinement in the riding model approximation, with *U*(H) set to 1.2 to 1.5*U*(C). The final difference Fourier map had a peak in the vicinity of the C13 and C14 atoms.

### Crystal data

**Table:**

[Al(C_5_H_7_O_3_)_3_]	*Z* = 2
*M**_r_* = 372.30	*F*(000) = 392
Triclinic, *P*1	*D*_x_ = 1.350 Mg m^−^^3^
Hall symbol: -P 1	Mo *K*α radiation, λ = 0.71073 Å
*a* = 6.476 (1) Å	Cell parameters from 712 reflections
*b* = 9.986 (2) Å	θ = 2.5–25.9°
*c* = 14.368 (2) Å	µ = 0.15 mm^−^^1^
α = 90.478 (2)°	*T* = 293 K
β = 92.229 (2)°	Block, yellow
γ = 99.337 (2)°	0.5 × 0.4 × 0.2 mm
*V* = 916.1 (2) Å^3^	

### Data collection

**Table:**

Bruker SMART APEX diffractometer	3207 independent reflections
Radiation source: fine-focus sealed tube	2304 reflections with *I* > 2σ(*I*)
graphite	*R*_int_ = 0.041
ω scans	θ_max_ = 25.0°, θ_min_ = 1.4°
Absorption correction: multi-scan *SADABS* (Sheldrick, 1996)	*h* = −7→7
*T*_min_ = 0.927, *T*_max_ = 0.970	*k* = −11→11
8801 measured reflections	*l* = −17→17

### Refinement

**Table:**

Refinement on *F*^2^	Primary atom site location: structure-invariant direct methods
Least-squares matrix: full	Secondary atom site location: difference Fourier map
*R*[*F*^2^ > 2σ(*F*^2^)] = 0.068	Hydrogen site location: inferred from neighbouring sites
*wR*(*F*^2^) = 0.189	H-atom parameters constrained
*S* = 1.02	*w* = 1/[σ^2^(*F*_o_^2^) + (0.0907*P*)^2^ + 1.477*P*] where *P* = (*F*_o_^2^ + 2*F*_c_^2^)/3
3207 reflections	(Δ/σ)_max_ = 0.001
232 parameters	Δρ_max_ = 1.26 e Å^−^^3^
0 restraints	Δρ_min_ = −0.28 e Å^−^^3^

### Fractional atomic coordinates and isotropic or equivalent isotropic displacement parameters (Å^2^)

**Table:**

	*x*	*y*	*z*	*U*_iso_*/*U*_eq_	
Al1	0.61876 (18)	0.34609 (11)	0.25756 (8)	0.0287 (3)	
O1	0.8373 (4)	0.4902 (2)	0.29494 (17)	0.0298 (6)	
O2	1.0420 (4)	0.6906 (3)	0.2872 (2)	0.0407 (7)	
O3	0.4846 (4)	0.4644 (3)	0.18716 (19)	0.0348 (7)	
O4	0.7671 (4)	0.2253 (2)	0.32512 (17)	0.0279 (6)	
O5	0.8034 (4)	0.0612 (3)	0.42532 (19)	0.0388 (7)	
O6	0.4689 (4)	0.3726 (2)	0.36122 (18)	0.0317 (6)	
O7	0.7870 (4)	0.3167 (3)	0.15592 (17)	0.0342 (7)	
O8	0.9151 (5)	0.1916 (3)	0.0516 (2)	0.0472 (8)	
O9	0.4156 (4)	0.1989 (3)	0.21949 (19)	0.0375 (7)	
C1	1.1890 (6)	0.6352 (5)	0.3464 (3)	0.0419 (10)	
H1A	1.3137	0.7011	0.3560	0.063*	
H1B	1.2235	0.5551	0.3176	0.063*	
H1C	1.1283	0.6125	0.4052	0.063*	
C2	0.8676 (6)	0.6084 (4)	0.2614 (3)	0.0302 (9)	
C3	0.7318 (7)	0.6615 (4)	0.1987 (3)	0.0364 (10)	
H3	0.7674	0.7509	0.1799	0.044*	
C4	0.5509 (6)	0.5875 (4)	0.1646 (3)	0.0324 (9)	
C5	0.4062 (7)	0.6453 (4)	0.0957 (3)	0.0418 (11)	
H5A	0.2692	0.6377	0.1203	0.063*	
H5B	0.3978	0.5957	0.0378	0.063*	
H5C	0.4602	0.7391	0.0854	0.063*	
C6	0.9613 (7)	0.0242 (5)	0.3656 (3)	0.0423 (10)	
H6A	1.0342	−0.0400	0.3968	0.063*	
H6B	1.0590	0.1039	0.3518	0.063*	
H6C	0.8953	−0.0157	0.3088	0.063*	
C7	0.7055 (6)	0.1615 (4)	0.3967 (3)	0.0280 (8)	
C9	0.4357 (6)	0.2936 (4)	0.4312 (3)	0.0317 (9)	
C8	0.5413 (6)	0.1888 (4)	0.4513 (3)	0.0336 (9)	
H8	0.5038	0.1345	0.5021	0.040*	
C10	0.2692 (7)	0.3274 (4)	0.4948 (3)	0.0433 (11)	
H10A	0.1454	0.3372	0.4583	0.065*	
H10B	0.3205	0.4109	0.5279	0.065*	
H10C	0.2364	0.2557	0.5385	0.065*	
C11	1.0873 (7)	0.2966 (5)	0.0492 (3)	0.0464 (11)	
H11A	1.1911	0.2695	0.0104	0.070*	
H11B	1.1462	0.3157	0.1112	0.070*	
H11C	1.0424	0.3766	0.0244	0.070*	
C12	0.7677 (7)	0.2051 (5)	0.1104 (3)	0.0380 (10)	
C13	0.5996 (7)	0.0964 (4)	0.1146 (3)	0.0403 (10)	
H13	0.6048	0.0183	0.0796	0.048*	
C14	0.4322 (7)	0.1000 (4)	0.1665 (3)	0.0379 (10)	
C15	0.2460 (7)	−0.0173 (4)	0.1643 (3)	0.0434 (11)	
H15A	0.1178	0.0182	0.1545	0.065*	
H15B	0.2434	−0.0639	0.2225	0.065*	
H15C	0.2605	−0.0795	0.1145	0.065*	

### Atomic displacement parameters (Å^2^)

**Table:**

	*U*^11^	*U*^22^	*U*^33^	*U*^12^	*U*^13^	*U*^23^
Al1	0.0348 (7)	0.0243 (6)	0.0268 (6)	0.0046 (5)	0.0003 (5)	0.0002 (4)
O1	0.0298 (14)	0.0295 (14)	0.0295 (14)	0.0021 (11)	0.0035 (11)	−0.0032 (11)
O2	0.0372 (17)	0.0351 (15)	0.0484 (18)	0.0012 (13)	0.0016 (14)	0.0019 (13)
O3	0.0390 (16)	0.0275 (14)	0.0395 (16)	0.0099 (12)	0.0002 (13)	0.0030 (12)
O4	0.0289 (14)	0.0320 (14)	0.0233 (13)	0.0055 (11)	0.0028 (11)	0.0030 (11)
O5	0.0450 (17)	0.0441 (17)	0.0313 (15)	0.0179 (14)	0.0079 (13)	0.0078 (12)
O6	0.0277 (14)	0.0289 (14)	0.0385 (16)	0.0035 (11)	0.0059 (12)	0.0016 (12)
O7	0.0491 (17)	0.0345 (15)	0.0217 (14)	0.0136 (13)	0.0050 (12)	−0.0011 (11)
O8	0.0407 (18)	0.058 (2)	0.0421 (18)	0.0050 (15)	0.0080 (14)	−0.0004 (15)
O9	0.0457 (17)	0.0283 (14)	0.0366 (16)	0.0027 (12)	−0.0069 (13)	−0.0013 (12)
C1	0.030 (2)	0.051 (3)	0.044 (3)	0.0045 (19)	0.0002 (19)	−0.001 (2)
C2	0.030 (2)	0.027 (2)	0.032 (2)	−0.0037 (16)	0.0129 (17)	−0.0102 (16)
C3	0.047 (3)	0.027 (2)	0.037 (2)	0.0094 (18)	0.0084 (19)	0.0068 (17)
C4	0.040 (2)	0.033 (2)	0.028 (2)	0.0124 (18)	0.0097 (17)	−0.0013 (16)
C5	0.055 (3)	0.037 (2)	0.038 (2)	0.022 (2)	0.004 (2)	0.0085 (18)
C6	0.044 (3)	0.047 (3)	0.041 (2)	0.023 (2)	0.006 (2)	0.007 (2)
C7	0.030 (2)	0.0283 (19)	0.0240 (19)	0.0028 (16)	−0.0050 (16)	0.0005 (15)
C9	0.029 (2)	0.029 (2)	0.034 (2)	−0.0040 (16)	0.0020 (17)	−0.0061 (17)
C8	0.038 (2)	0.035 (2)	0.026 (2)	−0.0002 (18)	0.0076 (17)	0.0030 (16)
C10	0.039 (2)	0.044 (2)	0.047 (3)	0.002 (2)	0.021 (2)	−0.006 (2)
C11	0.030 (2)	0.074 (3)	0.036 (2)	0.008 (2)	0.0128 (19)	0.003 (2)
C12	0.044 (2)	0.058 (3)	0.0174 (19)	0.026 (2)	0.0062 (17)	0.0084 (18)
C13	0.058 (3)	0.022 (2)	0.039 (2)	0.0031 (19)	−0.007 (2)	−0.0040 (17)
C14	0.049 (3)	0.036 (2)	0.030 (2)	0.0126 (19)	−0.0016 (19)	0.0033 (18)
C15	0.051 (3)	0.033 (2)	0.044 (3)	−0.001 (2)	0.009 (2)	−0.0113 (19)

### Geometric parameters (Å, °)

**Table:**

Al1—O1	1.905 (3)	C4—C5	1.518 (6)
Al1—O3	1.859 (3)	C5—H5A	0.9600
Al1—O4	1.909 (3)	C5—H5B	0.9600
Al1—O6	1.849 (3)	C5—H5C	0.9600
Al1—O7	1.904 (3)	C6—H6A	0.9600
Al1—O9	1.869 (3)	C6—H6B	0.9600
O1—C2	1.267 (5)	C6—H6C	0.9600
O2—C2	1.322 (4)	C7—C8	1.406 (5)
O2—C1	1.432 (5)	C9—C8	1.367 (6)
O3—C4	1.284 (5)	C9—C10	1.517 (5)
O4—C7	1.258 (4)	C8—H8	0.9300
O5—C7	1.329 (4)	C10—H10A	0.9600
O5—C6	1.450 (5)	C10—H10B	0.9600
O6—C9	1.286 (5)	C10—H10C	0.9600
O7—C12	1.273 (5)	C11—H11A	0.9600
O8—C12	1.322 (5)	C11—H11B	0.9600
O8—C11	1.403 (5)	C11—H11C	0.9600
O9—C14	1.263 (5)	C12—C13	1.411 (6)
C1—H1A	0.9600	C13—C14	1.345 (6)
C1—H1B	0.9600	C13—H13	0.9300
C1—H1C	0.9600	C14—C15	1.538 (6)
C2—C3	1.401 (6)	C15—H15A	0.9600
C3—C4	1.352 (6)	C15—H15B	0.9600
C3—H3	0.9300	C15—H15C	0.9600
			
O6—Al1—O3	92.13 (12)	H5B—C5—H5C	109.5
O6—Al1—O9	90.45 (13)	O5—C6—H6A	109.5
O3—Al1—O9	91.19 (13)	O5—C6—H6B	109.5
O6—Al1—O1	91.76 (12)	H6A—C6—H6B	109.5
O3—Al1—O1	90.98 (12)	O5—C6—H6C	109.5
O9—Al1—O1	176.85 (13)	H6A—C6—H6C	109.5
O6—Al1—O7	176.44 (13)	H6B—C6—H6C	109.5
O3—Al1—O7	90.98 (12)	O4—C7—O5	118.6 (3)
O9—Al1—O7	91.22 (13)	O4—C7—C8	125.3 (3)
O1—Al1—O7	86.45 (12)	O5—C7—C8	116.1 (3)
O6—Al1—O4	90.71 (12)	O6—C9—C8	124.9 (4)
O3—Al1—O4	177.05 (13)	O6—C9—C10	114.6 (3)
O9—Al1—O4	89.59 (12)	C8—C9—C10	120.5 (4)
O1—Al1—O4	88.13 (12)	C9—C8—C7	121.4 (3)
O7—Al1—O4	86.16 (12)	C9—C8—H8	119.3
C2—O1—Al1	126.1 (3)	C7—C8—H8	119.3
C2—O2—C1	116.9 (3)	C9—C10—H10A	109.5
C4—O3—Al1	129.5 (3)	C9—C10—H10B	109.5
C7—O4—Al1	125.6 (2)	H10A—C10—H10B	109.5
C7—O5—C6	116.8 (3)	C9—C10—H10C	109.5
C9—O6—Al1	127.3 (2)	H10A—C10—H10C	109.5
C12—O7—Al1	123.6 (3)	H10B—C10—H10C	109.5
C12—O8—C11	117.6 (4)	O8—C11—H11A	109.5
C14—O9—Al1	129.2 (3)	O8—C11—H11B	109.5
O2—C1—H1A	109.5	H11A—C11—H11B	109.5
O2—C1—H1B	109.5	O8—C11—H11C	109.5
H1A—C1—H1B	109.5	H11A—C11—H11C	109.5
O2—C1—H1C	109.5	H11B—C11—H11C	109.5
H1A—C1—H1C	109.5	O7—C12—O8	117.0 (4)
H1B—C1—H1C	109.5	O7—C12—C13	126.0 (4)
O1—C2—O2	118.0 (4)	O8—C12—C13	116.9 (4)
O1—C2—C3	125.9 (3)	C14—C13—C12	123.3 (4)
O2—C2—C3	116.1 (3)	C14—C13—H13	118.4
C4—C3—C2	122.4 (4)	C12—C13—H13	118.4
C4—C3—H3	118.8	O9—C14—C13	122.9 (4)
C2—C3—H3	118.8	O9—C14—C15	115.5 (4)
O3—C4—C3	124.0 (4)	C13—C14—C15	121.6 (4)
O3—C4—C5	114.0 (4)	C14—C15—H15A	109.5
C3—C4—C5	122.0 (4)	C14—C15—H15B	109.5
C4—C5—H5A	109.5	H15A—C15—H15B	109.5
C4—C5—H5B	109.5	C14—C15—H15C	109.5
H5A—C5—H5B	109.5	H15A—C15—H15C	109.5
C4—C5—H5C	109.5	H15B—C15—H15C	109.5
H5A—C5—H5C	109.5		
			
O6—Al1—O1—C2	−102.8 (3)	C1—O2—C2—C3	−176.1 (3)
O3—Al1—O1—C2	−10.6 (3)	O1—C2—C3—C4	−2.0 (6)
O7—Al1—O1—C2	80.3 (3)	O2—C2—C3—C4	178.1 (4)
O4—Al1—O1—C2	166.6 (3)	Al1—O3—C4—C3	−8.0 (6)
O6—Al1—O3—C4	102.4 (3)	Al1—O3—C4—C5	172.5 (3)
O9—Al1—O3—C4	−167.1 (3)	C2—C3—C4—O3	1.4 (6)
O1—Al1—O3—C4	10.6 (3)	C2—C3—C4—C5	−179.1 (4)
O7—Al1—O3—C4	−75.8 (3)	Al1—O4—C7—O5	166.7 (2)
O6—Al1—O4—C7	22.3 (3)	Al1—O4—C7—C8	−14.6 (5)
O9—Al1—O4—C7	−68.1 (3)	C6—O5—C7—O4	−6.2 (5)
O1—Al1—O4—C7	114.0 (3)	C6—O5—C7—C8	175.0 (3)
O7—Al1—O4—C7	−159.4 (3)	Al1—O6—C9—C8	14.4 (5)
O3—Al1—O6—C9	158.5 (3)	Al1—O6—C9—C10	−167.3 (3)
O9—Al1—O6—C9	67.3 (3)	O6—C9—C8—C7	2.3 (6)
O1—Al1—O6—C9	−110.5 (3)	C10—C9—C8—C7	−175.8 (4)
O4—Al1—O6—C9	−22.3 (3)	O4—C7—C8—C9	−1.8 (6)
O3—Al1—O7—C12	−110.4 (3)	O5—C7—C8—C9	176.9 (3)
O9—Al1—O7—C12	−19.2 (3)	Al1—O7—C12—O8	−169.6 (2)
O1—Al1—O7—C12	158.7 (3)	Al1—O7—C12—C13	12.8 (5)
O4—Al1—O7—C12	70.3 (3)	C11—O8—C12—O7	4.5 (5)
O6—Al1—O9—C14	−157.2 (3)	C11—O8—C12—C13	−177.6 (4)
O3—Al1—O9—C14	110.6 (3)	O7—C12—C13—C14	2.2 (7)
O7—Al1—O9—C14	19.6 (3)	O8—C12—C13—C14	−175.4 (4)
O4—Al1—O9—C14	−66.5 (3)	Al1—O9—C14—C13	−11.6 (6)
Al1—O1—C2—O2	−171.6 (2)	Al1—O9—C14—C15	168.8 (3)
Al1—O1—C2—C3	8.6 (5)	C12—C13—C14—O9	−3.4 (7)
C1—O2—C2—O1	4.1 (5)	C12—C13—C14—C15	176.2 (4)
